# Fournier's Gangrene Associated With Untreated Crohn’s Disease in a Male Patient: A Case Report and Review of the Literature

**DOI:** 10.7759/cureus.67515

**Published:** 2024-08-22

**Authors:** Nicos Kritharides, Marios Fouzas, Afroditi Stoupa, Spyridoula Sakkoula, Loukia Prounia - Alexopoulou, Georgios Rallis, Aikaterini Leventi

**Affiliations:** 1 Department of Surgery, General Hospital of Athens "Elpis", Athens, GRC; 2 Department of Surgical Oncology, Agios Savvas Anti-Cancer Oncology Hospital of Athens, Athens, GRC

**Keywords:** case report, inflammatory bowel disease, necrotizing fasciitis, crohn disease, fournier gangrene

## Abstract

Crohn’s disease is a chronic idiopathic inflammatory bowel disease that can affect any part of the gastrointestinal tract. Perianal symptoms are seen in one-third of Crohn's disease cases, with perianal abscesses leading to Fournier's gangrene being extremely rare. Herein, we discuss an interesting case of a male patient with Fournier’s gangrene as a result of untreated Crohn’s disease.

A 51-year-old male presented to the emergency department with a perianal abscess and cellulitis of the perineum. Examination under general anesthesia (EUA) of the rectum and incision and drainage (I&D) of the abscess were performed urgently, leading to the diagnosis of Fournier’s gangrene. Subsequent investigations revealed that the causative factor was a previously diagnosed but untreated Crohn’s disease. The comprehensive treatment plan included fecal diversion, regular surgical debridement, negative pressure wound therapy, antibiotics administration, and perineal reconstruction. After a 37-day hospital stay, the patient was discharged in good clinical condition and referred to a specialized gastroenterologist for further treatment. A year later, he underwent an ileocecal resection with ileocolic anastomosis.

In rare circumstances, Crohn’s disease may manifest solely through perianal symptoms and, even more rarely, as Fournier’s gangrene. It is crucial for clinicians to be aware of this manifestation for early diagnosis and prompt treatment.

Maintaining a high level of suspicion, achieving early diagnosis, implementing prompt resuscitation, and adopting a multidisciplinary approach within specialized medical centers are crucial factors for effective management in these cases.

## Introduction

Crohn’s disease (CD) affects the gastrointestinal tract, with prevalent symptoms including abdominal pain, blood or mucus diarrhea, fever, and bowel obstruction. [1] The annual incidence ranges from three to 20 cases per 100,000 individuals. Notably, it exhibits a higher prevalence in North America and Western Europe, with a greater incidence in women compared to men and in Ashkenazi Jews compared to other ethnicities [2]. In Europe, an estimated 0.2% of the population suffers from inflammatory bowel disease (IBD). A recent study in Greece revealed that 0.15% of young males are diagnosed with IBD, with 53.4% having CD and 46.4% having ulcerative colitis (UC) [3,4].

Fournier’s gangrene (FG) is a necrotizing soft tissue disease, named after the famous venereologist Jean Alfred Fournier, who initially described it in 1883. This uncommon disease afflicts 1.6 in 100,000 men annually, with an average patient age of 50.9 years. Although women can also experience FG, the male-to-female ratio is 10:1. Infections affecting the urogenital tract, the anorectal area, or the skin around the genitals contribute to the pathogenesis in approximately 95% of cases [5]. Immunocompromised patients, including those with diabetes, obesity, and malignancies, are at a heightened risk of developing FG [5].

The association between CD and FG has been reported in the literature through a limited number of case reports. Specifically, six additional cases of necrotizing fasciitis linked to CD have been documented. In four of these cases, FG emerged as the initial presentation of CD, like the case we present in the current article [6-11]. The underlying cause of this connection has not been firmly established. Some authors have attributed the presence of enterocutaneous fistulas and the use of immunosuppression therapies for CD as potential predisposing factors [6,12]. The high morbidity and mortality of such cases demand prompt and well-designed management.

## Case presentation

A 51-year-old male patient presented to the emergency department (ED) with perianal swelling, pain, and fever. He reported the onset of his symptoms a few days before his admission. The patient was a heavy smoker with a history of idiopathic hypertension and chronic severe but asymptomatic pericardial effusion. Additionally, he mentioned undergoing an investigation for an episode of melena three years ago but without receiving a specific diagnosis. Interestingly, his sister was diagnosed with UC at the age of 40.

Upon digital rectal examination, a perianal abscess of the right buttock was identified, along with cellulitis that extended to the perineum and right gluteal region. Laboratory tests revealed elevated inflammatory markers (Table 1).

**Table 1:** Laboratory tests upon admission showed elevated inflammatory markers

Laboratory Test	Result	Reference Value
White blood cells	24.010 k/μL	4.0-11.0 k/μL
Neutrophils	92%	45-85%
C-reactive protein	37.25 mg/dL	< 0.30 mg/dL

Following informed consent, the patient underwent an examination under anesthesia (EUA) in the operating theater. The EUA revealed a perianal abscess accompanied by cellulitis of the right buttock. Incision and drainage (I&D) of the abscess were performed, and pus samples were collected for further microbiology examination. Upon returning to the ward, and further questioning, the patient shared gastroscopy and colonoscopy reports indicating terminal ileitis and a diagnosis of IBD dating back two years. Histology results obtained during these investigations confirmed CD. Considering himself asymptomatic, except for the isolated episode of melena, and burdened by an extreme workload, he had neglected to seek further treatment. Nevertheless, upon revisiting his symptomatology, he admitted to experiencing atypical dyspeptic symptoms, bloating, and sporadically watery diarrheas.

**Figure 1:**
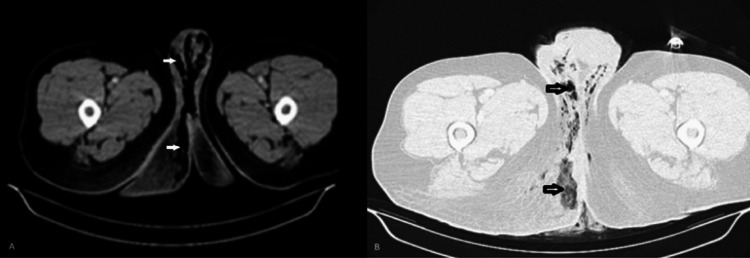
A. Axial CT scan of the perianal region. The white arrows indicate the presence of diffuse gas within the perineum and soft tissues. B. Axial CT scan of the perianal region (lung window). The arrows indicate the presence of diffuse gas within the perineum and soft tissues.

Furthermore, 48 hours following a transient improvement, he experienced a gradual deterioration with sepsis recurrence, persistent drainage of malodorous pus from the perianal wound, and, finally, spreading of the cellulitis from the perineum to the right buttock by the fifth postoperative day. Due to these findings, an emergency computed tomography (CT) scan was performed, which revealed diffuse gas within the perineum and soft tissues (Figure 1).

**Figure 2:**
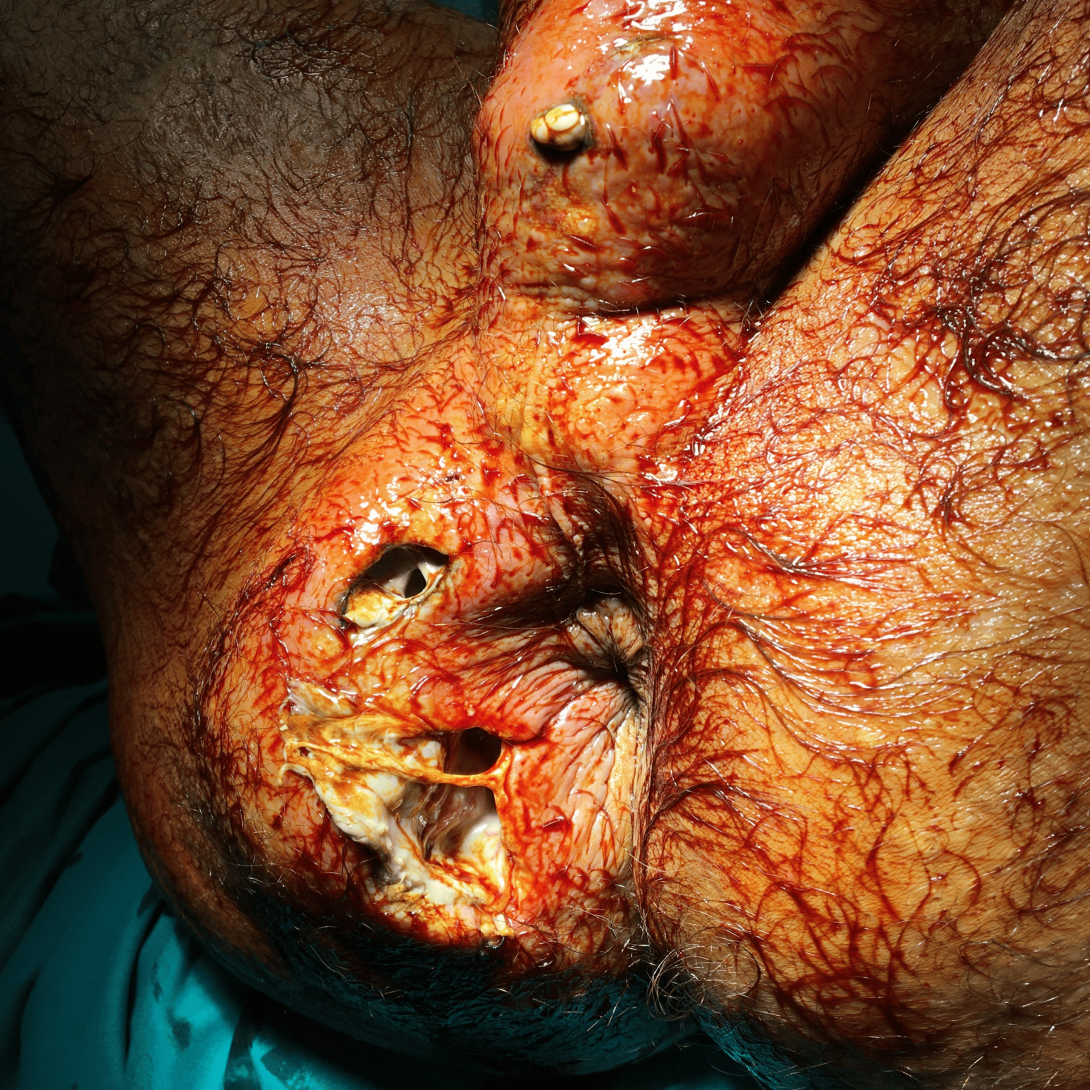
Necrotizing soft tissue infection of the right buttock and perineum, resulting from a high transsphincteric fistula, which communicated with a posterior horseshoe abscess and ended up with an internal opening at the 6 o'clock position. The necrotizing soft tissue infection had extended to the base of the scrotum.

**Figure 3:**
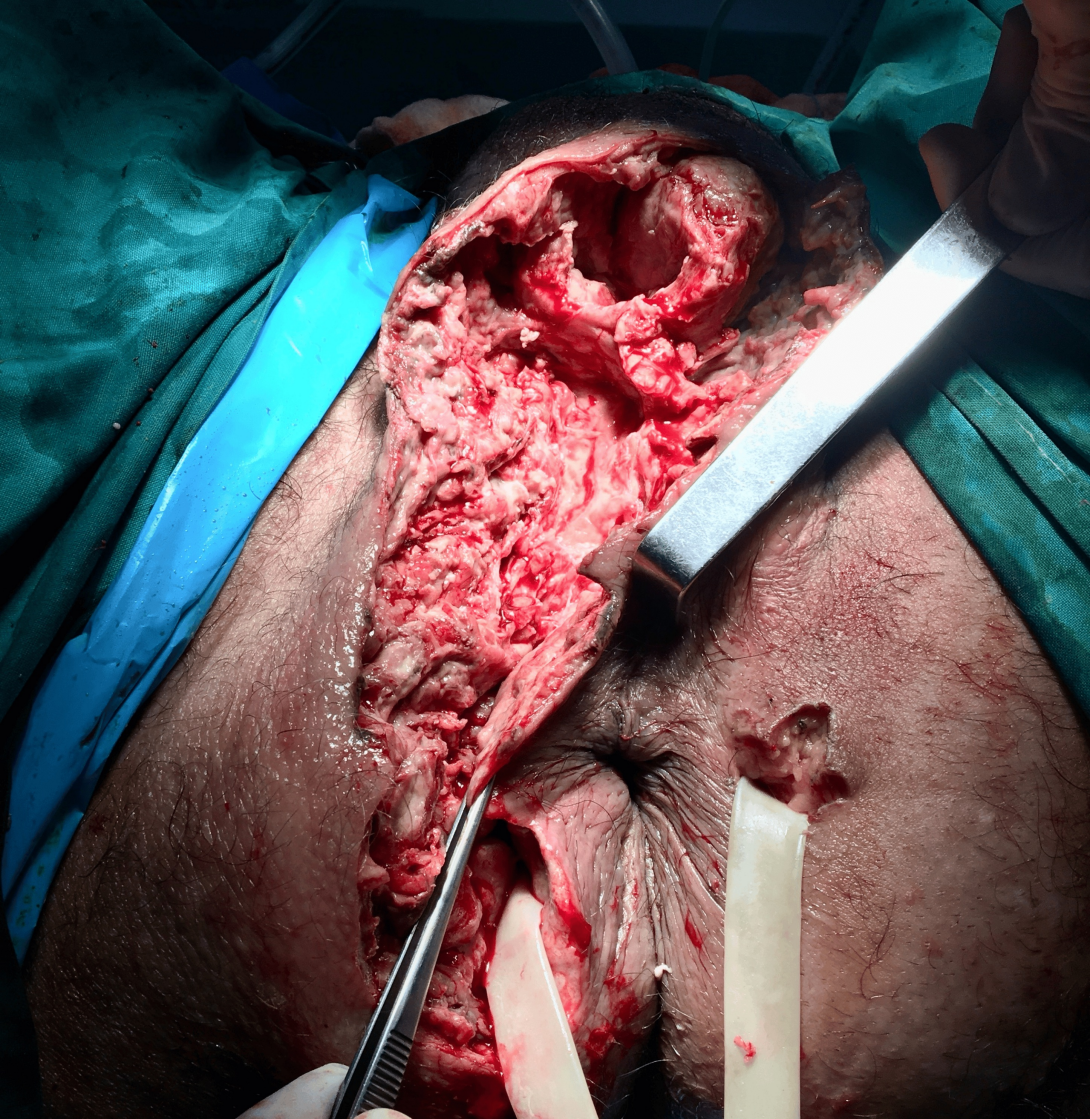
Healthy tissue following surgical debridement and drainage of the horseshoe abscess from both the right and left ischiorectal fossa. A Penrose drain was placed to facilitate the drainage of the fistula.

Immediately, the patient was referred to the colorectal surgeon of our department for further evaluation and treatment and underwent an operation the same day. Intraoperative findings confirmed the diagnosis of FG due to a high transphincteric fistula communicating with a posterior horseshoe abscess and originating from an internal opening at the 6 o'clock position (Figures 2, 3). At the same time, the necrotizing soft tissue infection had extended to the base of the scrotum, prompting the involvement of a urologist for surgical debridement around the right testicle. Tissue and pus specimens were collected for histology and culture testing.

**Figure 4:**
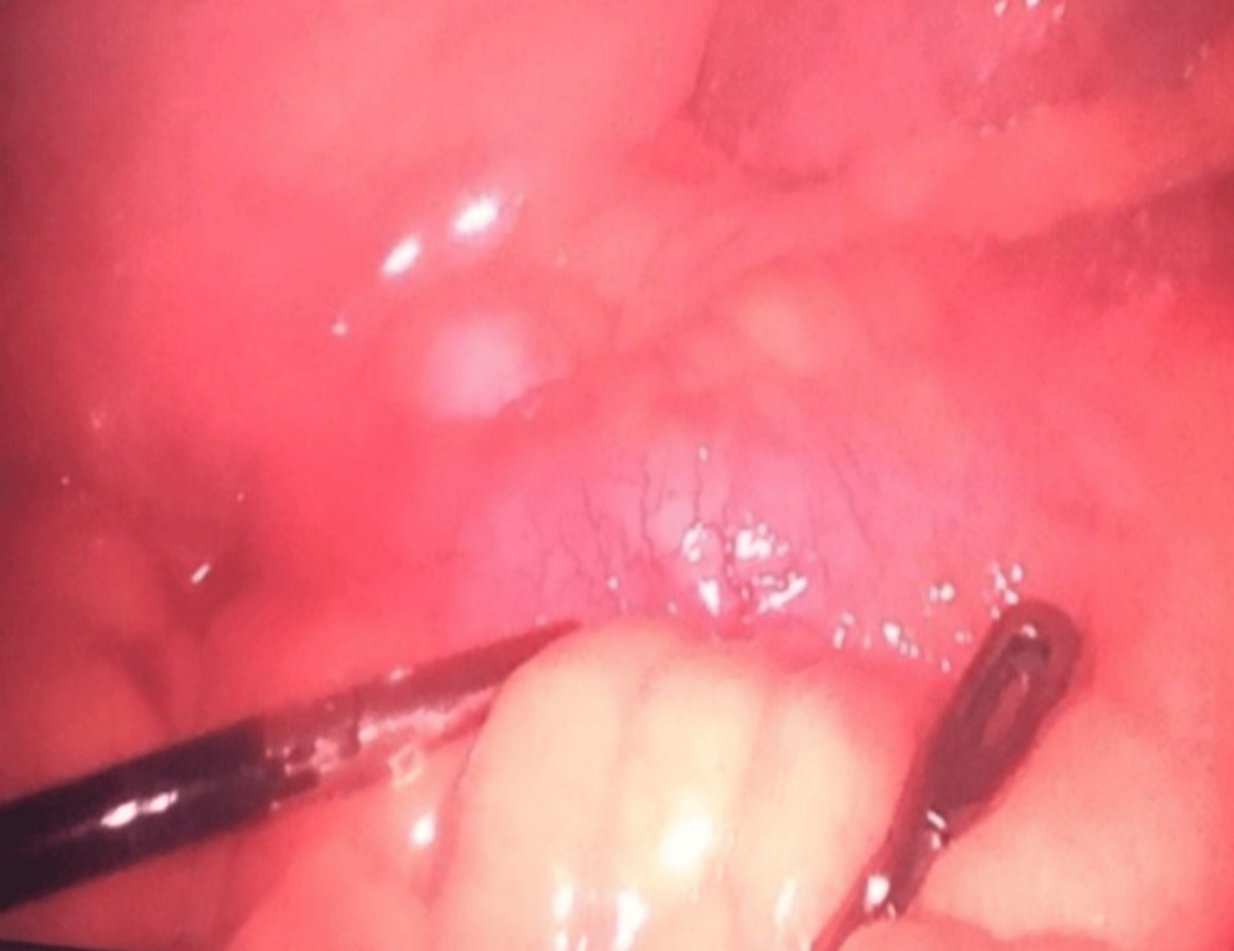
Laparoscopic view showing active terminal ileal disease with fat wrapping, indicative of Crohn's disease.

**Figure 5:**
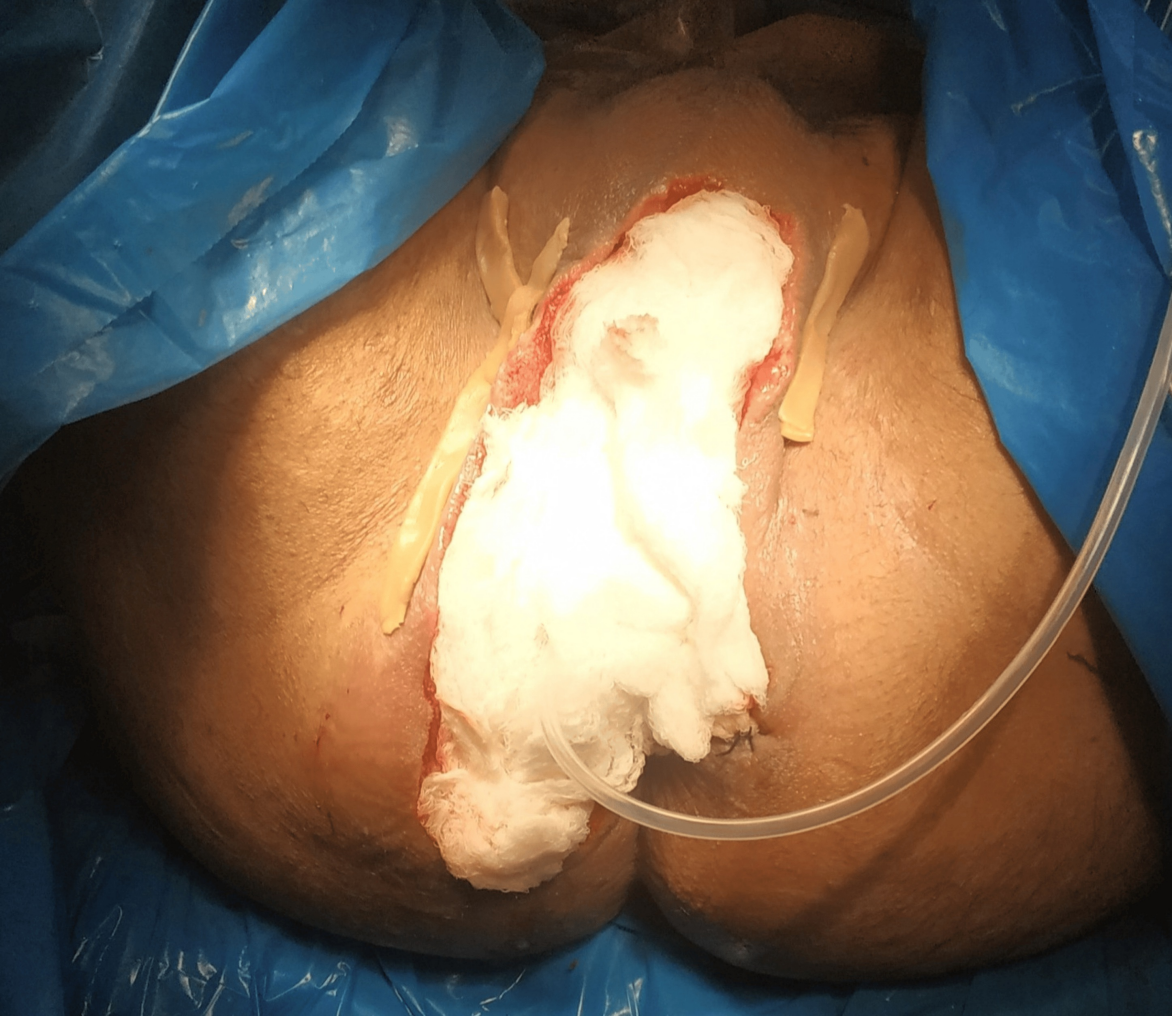
Application of a negative pressure wound therapy system (VAC) to the surgical wound after debridement to facilitate wound healing. VAC, vacuum-assisted closure

After the second operation, the patient was hemodynamically stable, remained afebrile, and presented improvement in inflammatory markers. A planned “second look” operation was conducted 48 hours later for additional surgical debridement. Given the necessity for fecal diversion to facilitate wound healing and divert the bowel away from the partially stenosed terminal ileum (TI), a diagnostic laparoscopy was performed during this operation, and a loop ileostomy was created. Although the initial CT scan did not show any evidence of prestenotic dilatation, as it was performed to assess perianal sepsis, further discussion with radiologists - after the diagnosis was clarified - revealed thickening of the bowel wall in the TI with narrowing of the lumen. The laparoscopy confirmed active terminal ileal disease with fat wrapping in the last 40 cm of TI, and an area with healthy bowel proximal to the disease was selected for the ileostomy formation (Figure 4). The debridement was successfully carried out, reaching healthy tissues and halting the advancement of inflammation along the fascia. Upon completing the operation, a negative pressure system (vacuum-assisted closure [VAC] of a wound) was applied to the surgical wound (Figure 5). Due to the extensive surgical wound, the patient returned to the operating room approximately every 72 hours for repeated surgical debridement and VAC dressing changes under general anesthesia.

During his hospitalization and once stabilized, a comprehensive assessment of the extraintestinal manifestations of his CD was conducted. Interestingly, he revealed a diagnosis of pericardial effusion during a routine cardiological screening back in 2009, a finding confirmed by subsequent imaging tests. In the current hospitalization, cardiologists identified a chronic pericardial effusion, although its underlying cause remained unknown. They recommended a water restriction and the initiation of antihypertensive medication (Irbesartan). Furthermore, he reported a prior hospitalization two years ago due to iridocyclitis in his right eye, which was successfully treated with cortisone. However, the ophthalmologist's examination during his current admission did not reveal any pathological findings. Concluding the thorough investigation, no additional extraintestinal manifestations were identified. The Crohn’s Disease Activity Index (CDAI) score was calculated at 237, revealing moderately active disease at the current state [13]. At this point, it is essential to highlight that during his hospital stay, he received enteral nutrition with high protein content to cover his anabolic needs.

Throughout, the patient received various antibiotic therapies based on antibiograms. Upon admission, he was initially treated with ciprofloxacin and metronidazole empirically. Pus culture from the EUA identified *Enterococcus faecalis*. Due to the patient's deterioration in the early postoperative days, linezolid was added to the antimicrobial regimen. During the second operation, wound cultures revealed *Staphylococcus aureus* resistant to ciprofloxacin. Consequently, the infectious disease specialist recommended discontinuing ciprofloxacin, metronidazole, and linezolid, and starting treatment with piperacillin/tazobactam and daptomycin based on sensitivities. Seven days later, repeat wound culture identified *Klebsiella pneumoniae* with multi-drug resistance. Given these results and considering the planned perianal reconstruction, the Infectious Disease specialist advised discontinuing piperacillin/tazobactam and initiating therapy with tigecycline and colistin based on the antibiogram for a duration of seven days.

Additionally, during his hospital stay, an MR enterography was performed to thoroughly assess the extent of his CD. This examination revealed that the distal ileum, from the ileocecal junction to the ileostomy (length 40-50 cm), exhibited wall thickening and incomplete lumen expansion. These findings were consistent with chronic fibrotic changes.

**Figure 6:**
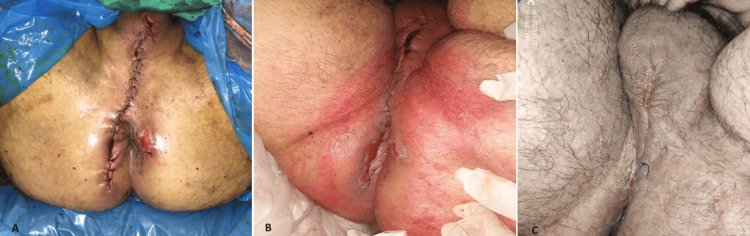
A. The figure shows the outcome immediately after perianal trauma restoration using an advancement flap. B. Seven days after perianal trauma restoration. C. 27 days after perianal trauma restoration.

**Figure 7:**
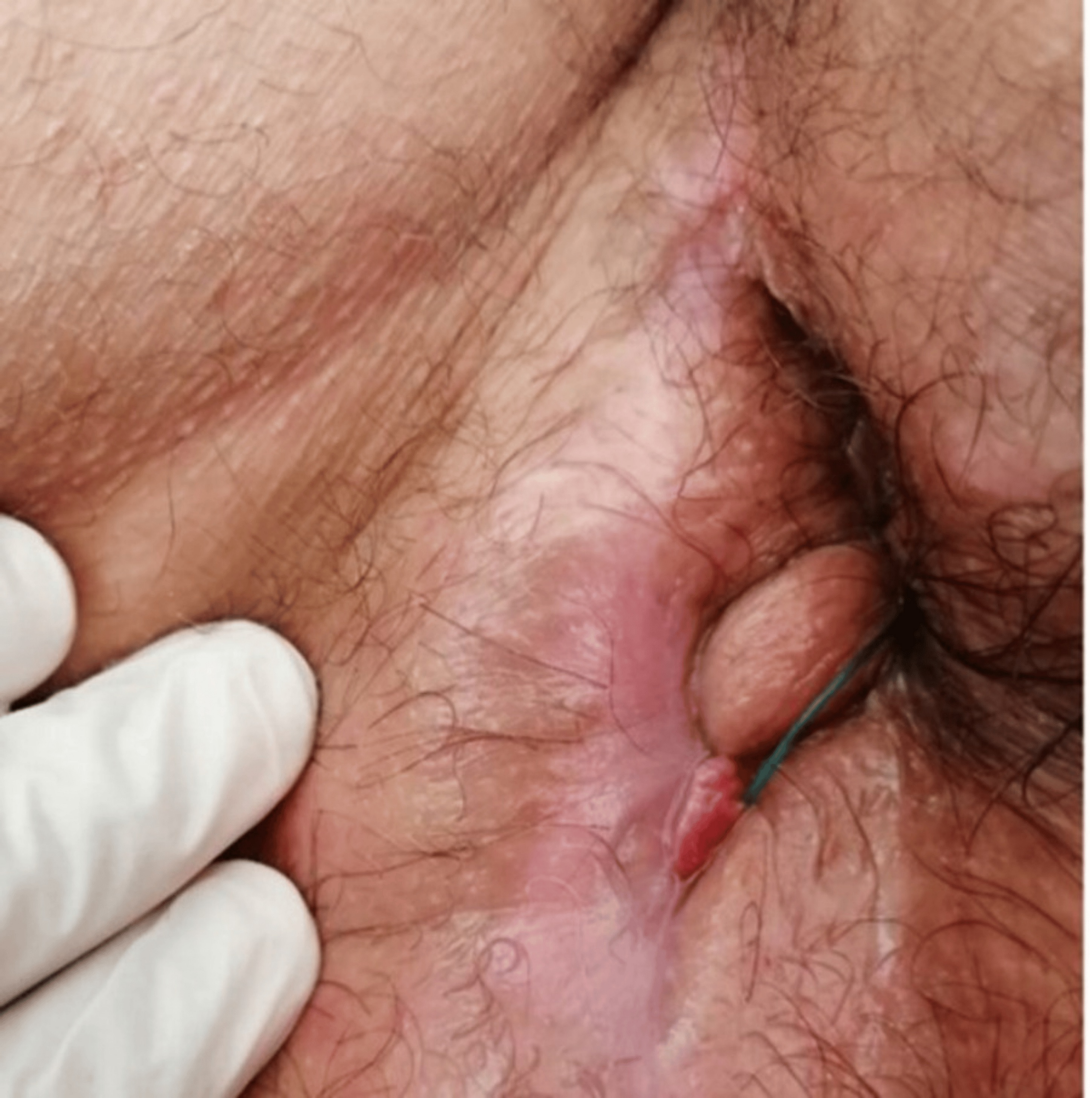
Loose seton in the transsphincteric fistula one month after the final EUA and closure of the complex fistula's blind tracts. EUA, examination under general anesthesia

A month after the initial operation, the perianal trauma was successfully restored using an advancement flap as a result of a collaborative effort with the help of a plastic surgeon (Figure 6). An upper gastrointestinal tract endoscopy uncovered CD involvement in the stomach and duodenum. The patient’s disease was classified as A2L4B1p according to the Montreal classification [14]. A week after perianal reconstruction, the patient was discharged in excellent clinical condition and promptly referred to a specialized gastroenterologist for close follow-up and further disease treatment. The patient commenced treatment of his disease with infliximab. Two months after discharge, and once the perineal wound had stabilized, an EUA was conducted, during which a loose seton was introduced to the transsphincteric fistula. Four months after this operation, an EUA was repeated. This examination involved the replacement of the loose seton, partial fistulectomy, and marsupialization of the blind tracts connected to the transsphincteric fistula. Ultimately, fistula track laser closure (FiLAC) procedure was performed to address the complex fistula’s blind tracts [15]. However, a shared decision was reached to not remove the seton placed in the high internal opening, as part of the treatment plan (Figure 7).

One year later, following successful disease control, we proceeded with stoma reversal, involving an ileocecectomy with ileocolic anastomosis. An anti-TNF agent was added to the treatment by the gastroenterologist, and MR enterography and colonoscopy were repeated before the ileostomy reversal to exclude active disease. In addition, the reversal of the ileostomy was combined with resection of the diseased bowel with an ileocecectomy and ileocolonic anastomosis. The decision to reverse the ileostomy was challenging due to the potential risk of perianal disease relapse. Since then, there has been no recurrence of perineal sepsis and no evidence of disease recurrence in the ileocolic anastomosis based on repeated colonoscopies. However, the blind tracts persisted, and the loose seton was kept in place with minimal output (Figure 7). The patient is under close follow-up, and his disease remains in remission with the administration of anti-TNF therapy, without evidence of clinical or endoscopic recurrence.

## Discussion

CD is a gastrointestinal condition characterized by chronic transmural inflammation with periods of activity and remission [16]. Its origin involves modifications in the bowel mucosa immune system, influenced by genetic and environmental factors. Common symptoms include chronic diarrhea, weight loss, and abdominal pain, with diagnosis requiring clinical evaluation, biochemical markers, and endoscopic, radiologic, and histological examinations [17]. The disease primarily affects the small intestine, especially the distal ileum (80% of cases), while in 30% of cases, it may initially present as a perianal disease [17]. Approximately 4-10% of patients have perianal fistulas at diagnosis, which can extend to nearby tissues such as the bladder, groin, or vagina [17,18]. Prompt surgical drainage is crucial for perianal abscesses, complemented by the placement of a loose seton in special circumstances and the administration of an appropriate antibiotic regimen [18]. In rare cases, as shown in this article, untreated perianal disease can rapidly progress to FG [6-11].

FG is a severe form of necrotizing fasciitis affecting the perianal region and external genital organs. It typically begins as cellulitis at the entry point, usually in the perineum or perianal region, and can rapidly spread to the skin and subcutaneous tissue, ultimately leading to severe infection, multiple organ failure, and death [19]. Symptoms and signs include pain, swelling, skin necrosis, and crepitus due to gas-forming bacteria. Sepsis signs such as fever, tachycardia, hypotension, and leukocytosis may also be present. FG is a polymicrobial infection commonly involving streptococci, staphylococci, Clostridia, Klebsiella, and Bacteroides [5,20]. In our patient, *Enterococcus faecalis*, *Staphylococcus aureus*, and *Klebsiella pneumoniae* were identified, while other cases linked to CD have reported organisms such as *Escherichia coli*, *Citrobacter *species, and *Candida albicans *[6,7,10,11]. The pathophysiology of necrotizing fasciitis involves cytokine cascade activation triggered by the bacterial infection, leading to endothelial damage, disrupted fibrinolysis, and thrombus formation in microvessels, causing tissue necrosis and rapid spread of infection ultimately leading to sepsis, multi-organ failure, and eventually death [5,20].

Risk factors include diabetes mellitus (20-70% of patients), immunosuppression, chemotherapy, alcohol consumption, and chronic corticosteroid use [20]. Diagnosis relies on clinical evaluation, laboratory tests, and imaging (ultrasonography, CT, MRI). Ultrasound also assists in testicular examination, which is usually unaffected in FG due to its distinct blood supply [5,20]. The Laboratory Risk Indicator for Necrotizing Fasciitis (LRINEC) can help differentiate necrotizing fasciitis from cellulitis and abscesses. With a total score of 13, a score of ≥6 raises suspicion of necrotizing fasciitis and the necessity of immediate intervention. In our case, the LRINEC score was 8, indicating a high risk for necrotizing soft tissue infection [21,22].

Treatment involves emergency surgical debridement under general anesthesia and broad-spectrum antibiotics such as second and third-generation cephalosporins combined with quinolones, aminoglycosides, nitroimidazole, and carbapenems [5,20]. Regular clinical examination and further debridement are crucial for prognosis.

We conducted an up-to-date literature search and identified six cases similar to ours [6-11]. In four of these cases, FG was reported as the initial manifestation of CD in patients with no known history of the latter condition, and in all situations, aggressive management with surgical debridement and broad-spectrum antibiotics was implemented [6-9,10]. In two instances, young male patients with an unremarkable medical history presented to the ED with common FG symptoms, such as pain, fever, swelling, and erythema extending to the perineum and scrotum [6,8]. They were diagnosed with FG and managed with debridement and antibiotic treatment [6,8]. Ileostomy was performed in both cases, and subsequent colonoscopy with typical findings of CD confirmed the diagnosis. In both cases, the patients’ course was uneventful, and they fully recovered after receiving appropriate therapy for their CD [6,8]. Interestingly, in one case, a fistula-in-ano (probably due to undiagnosed CD) was the primary trigger for FG [8], like our patient.

The first point we would like to highlight is the management of the patient described in our case study. Proper management involves aggressive resuscitation and treatment, encompassing strategies for acute perineal sepsis and severe exacerbations of CD. This includes prompt diagnosis, resuscitation with oxygen and fluids, administration of broad-spectrum antibiotics (effective against streptococci, staphylococci, anaerobes, and gram-negative bacteria), radical surgical intervention, repeated surgical debridement, creation of a diverting ileostomy, if necessary, intensive care monitoring, and eventually wound reconstruction. Moreover, early nutritional support is crucial for patient recovery [23]. Importantly, the treatment of such patients should take place in specialized medical centers. This is imperative because the comprehensive management of the combination of CD and perineal sepsis necessitates a re-evaluation of disease behavior before proceeding to the next steps of treatment.

Another crucial aspect demanding careful consideration is the role of ileostomy. In this case, we chose laparoscopic ileostomy over colostomy, a decision driven by the patient's confirmed terminal ileal disease. The decision to choose an ileostomy was made to prevent potential complications arising from TI stenosis. Although the stenosis had not yet manifested obstructive symptoms, its presence was detected in abdominal CT scans. A loop ileostomy is generally the preferred option for fecal diversion in cases of CD, as it has shown effectiveness when combined with medical treatment to achieve disease remission in selected cases [24].

One dilemma that we encountered after achieving disease remission was whether to proceed with ileostomy reversal, given the potential risk of perianal disease relapse. Fortunately, such a scenario did not occur. A systematic review and meta-analysis conducted in 2015 revealed that two-thirds of patients with perianal disease experienced clinical improvement with fecal diversion. However, among those patients showing clinical improvement, only one-third were deemed suitable candidates for bowel continuity restoration, and ultimately only 17% successfully underwent bowel restoration [24]. Despite the elevated risk of relapse, our patient maintains a good clinical condition, and his disease is currently in remission.

Furthermore, it is essential to address our patient's behavior regarding his disease. Despite a prior diagnosis of CD, he had never sought further medical support. Several studies have investigated the peculiarities on patients’ behavior post-IBD diagnosis. Chan et al.’s study on patients’ medication adherence after IBD diagnosis revealed a wide range of adherence, ranging from 7% to 72% [25]. One reason for this variability is the limited information provided by healthcare providers at the time of the initial diagnosis. Pittet et al. found that only 12.5% of patients were satisfied with the information they received at the time of diagnosis [26]. In another study, Lai et al. investigated the key drivers of patients’ decision-making after the diagnosis of IBD. They found that self-assessment of clinical condition and disease severity influenced patients’ attitude towards seeking medical support. During remission, patients tended to forget about their disease and medications. In contrast, they actively sought information and treatment options only during exacerbation. Additionally, some patients struggled to accept a chronic disease diagnosis, leading them to minimize their symptoms and delay seeking medical support. Individuals’ beliefs and knowledge, patient-provider relationships, social stigma, and healthcare system delays further contribute to delayed CD diagnosis and treatment [27].

Concerning our patient, the subtle nature of his symptoms prior to perineal sepsis, combined with his demanding workload and his personality, seems to be the main reasons for not seeking medical advice for his disease. Additionally, the diagnostic method (part of a common diagnostic protocol of melena investigation) may not have sufficiently alerted him to the severity and nature of his disease. Healthcare providers who first diagnose a patient with an IBD should dedicate time to provide all the necessary information and discuss with patients about their disease, treatment options, dangers of non-adherence to medication, and should also ensure close follow-up, especially in the first years after the diagnosis.

We believe that treatment strategies for concomitant FG and CD should be indeed based on the treatment protocols for each condition individually. However, managing both conditions simultaneously is more demanding and complex, necessitating specialized care. Decisions like the selection of the diverting ileostomy centrally to the impending terminal ileal stricture, the management of the fistula in the background of surgical debridement for perineal sepsis, and the coordination of surgical interventions with optimal IBD treatment demand specialized care with collaboration of a colorectal and emergency surgeon, infectious disease specialist, gastroenterologist, and plastic surgeon. Furthermore, as CD is a chronic condition, multidisciplinary decisions need to be made even after the resolution of the acute septic condition. For example, the timing of ileostomy reversal depends not only on the successful treatment of FG but also on the response to medical treatment for IBD. This approach ensures comprehensive care and careful re-evaluation of disease behavior before any further interventions.

The main limitation of our study is that all the information and treatment strategies are based on a limited number of cases due to the rarity of these cases. As a result, establishing generalized guidelines for proper management is challenging. However, all case reports described in the literature consistently agree and emphasize the importance of aggressive management and fecal diversion for the successful treatment. Our article further highlights the necessity of a multidisciplinary approach, the importance of regular evaluation of CD behavior before proceeding to the next therapeutic step, and the attitude of patients with IBD after diagnosis of their disease. This information can raise awareness among all medical specialties that encounter these patients, ensuring optimal care.

## Conclusions

CD manifests in various ways, and isolated perianal disease is no exception. On the other hand, numerous diseases can lead to FG, with CD being one of them. Although the association between the two is rare, heightened suspicion is crucial for early diagnosis. Effective management necessitates an aggressive, multidisciplinary approach, preferably in specialized medical centers, coupled with regular re-evaluation and close follow-up. Finally, emphasis should be placed on the proper guidance and regular supervision of patients upon the diagnosis of CD. Had our patient been properly informed, considering the severity of the initial diagnosis, progressing to this life-threatening condition might have been avoided.
